# Social affiliation is sufficient to provoke the partner-advantage

**DOI:** 10.1038/s41598-022-25052-1

**Published:** 2022-12-09

**Authors:** Chia-huei Tseng, Li Jingling, Miao Cheng

**Affiliations:** 1 Research Institute of Electrical Communication, Tohoku University, Sendai, Japan; 2 Graduate Institute of Biomedical Sciences, China Medical University, Taichung City, Taiwan; 3 NTT Communication Science Laboratories, NTT Corporation, Atsugi, Japan

**Keywords:** Human behaviour, Social behaviour

## Abstract

The partner-advantage is a type of identity-priority processing that we afford to a person with whom we perform a task together ^1^. The partner-advantage has been revealed by shortened reaction time (RT) and enhanced accuracy when participants learned to match a shape with an associated name. It is distinguished from other long-lasting and robust identity advantages (e.g., self-advantage and friend-advantage) by its instantaneous build-up and quick reduction; however, its characteristics and enabling factors remain unknown. The present study addresses these questions. In Experiment 1, we replicated the partner-advantage in a solo shape-name matching task (i.e., without a social component) in which other identity biases are usually reported. In Experiment 2, an absent partner (who did not appear physically) was sufficient to induce beneficial partner-related processing, with a temporary partner enjoying a benefit similar to that of significant others. In Experiment 3, an identity low in socially affiliated significance (e.g., another participant in the same experiment) did not automatically enjoy a priority bias. Taken together, our results suggest that the bias toward partners, similar to other known identity biases, does not require physical presence to build and maintain a referential advantage. The partner-advantage does not automatically extend to other social affiliations, and a joint task is not a pre-requisite to produce the bias. Our study offers new insights on identity-referential processing and its underlying mechanisms.

## Introduction

The most significant behavioral change during the Covid-19 pandemic has been an increase in online and remote activities. We are more likely to meet or work with those who we have never seen before but have to quickly establish the social identities of the group members (e.g. the partner, co-workers, boss, client) with minimum information (e.g. online ID, photo/image only). Without the usual in-person interaction and available social cues, the question of how information relevant to others is processed has become a focus of interest. In this study we focused on the “partner-advantage” which refers to the phenomenon that higher processing priority goes to events or stimuli associated with our partners compared to that to strangers^1^. Because many major behavioral changes can become part of a new norm (e.g., remote collaboration or work-from-home), the present study addresses a type of identity-related processing toward newly encountered co-workers and examines whether physical presence matters in initiating and sustaining the advantages associated with new co-workers.

The processing priority of a stimulus is modulated when the stimulus is attached to an identity. For example, an arbitrary object will become easier to remember^2^, to attend to^3^, and to identify if it is associated with “self”^4–6^. It has been proposed that a Self-Attention Network^7^ prioritizes the perceptual selection and attentional resources that endow self-related information on team members who are characterized as extensions of the self to the collective^8^. Similar effects have also been reported for significant identities such as best friends and mothers^5,6^; these are known to be more easily affected by experimental context than self-advantage is^6,9,10^.

“Self” and other significant identities emerge after years of long-term buildup, which has prompted some to argue that this information priority may be a simple reflection of familiarity. However, Cheng and Tseng^1^ tried to delineate the role of familiarity by introducing a socially significant identity (i.e., a co-actor in a joint task) without prior interaction history. Participants were introduced to a novel partner, with whom they had no opportunity for interaction during the experiment. In the task, participants were required to memorize associations between three shapes and three names: those of the participant, the partner, and a stranger. Identity prioritization over the stranger (e.g., faster RT, higher accuracy) was found to be similar for trials associated with the self and the newly introduced partner. That is, identity advantage can be observed with persons in low familiarity. This partner bias was established quickly within the first 45 trials and reduced or disappeared after approximately 90 trials. This temporal profile is distinct from the robust and long-lasting bias toward the self and significant others. In addition, the partner-advantage was reported only when the partner physically appeared in the same experiment room (whether performing the task together side-by-side simultaneously with the participant or only appearing physically briefly before the task), not when the partner was absent. Therefore, it is possible that the first impression from the physical appearance plays a critical role in forming such a partner-advantage. However, the necessary and sufficient conditions that produce this partner bias remain unclear, and it is unknown whether this bias is supported by mechanisms similar to those subserved by other identity processes.

The present study includes a joint task in which two participants perform a task together by sharing the trials (e.g., go/no-go trials). Other classic joint-action tasks (e.g., the joint Simon task^11^) have demonstrated that co-representation of a task by the two participants affects self/other processes. For example, in the standard (solo) Simon task, stimuli of two colors (e.g., blue and green) randomly appear at the left or right side of the screen, and a participant uses left and right buttons to indicate the color that appears on each trial. In the typical results, participants’ responses are affected by stimulus locations that are task-irrelevant: faster responses are observed when the response button and stimulus are on the same side (congruent) than when they are on opposite sides (incongruent). This congruency effect disappears when the participant must respond to only one color. Interestingly, when another participant joins and responds to the other color (i.e., joint Simon task), the congruency effect reappears^11^. This suggests that joint action facilitates self/other overlap, and the participant automatically represents the co-actor’s task goal and action. The co-actor’s representation creates a stimulus–response connection and interferes with the participant’s own task (e.g., it impedes performance when stimulus and response keys are on different sides). In the present study, we manipulate task setting, joint versus solo, to evaluate whether joint action is a crucial factor in generating a partner-advantage.

We examine the social nature of this effect by means of a shape-name association task with three identities: friend, partner, and stranger. By comparing performance on partner- and friend-associated trials versus stranger-associated trials, we are able to evaluate both the partner-advantage, as our main research focus, and the friend-advantage, as a reference for the partner-advantage. Because the previously reported identity priorities were mostly observed in a solo task, we first ask whether a social (joint) task is necessary to produce partner bias. Because other well-known biases (e.g. self, best friend, mother) can be retrieved without the physical presence of those target identities, we investigate whether the physical presence of a partner is required in partner bias. We also identify the boundary conditions of partner bias by asking whether other social affiliations also enjoy a similar endowment from “me” to “we”. By comparing these characteristics to previously reported identity advantages toward significant others, we hope to provide insights about the possible mechanisms of partner bias.

## Experiment 1: solo task

### Purpose

This experiment was intended to investigate whether a social joint task is necessary to establish a partner effect.

### Participants

The sample size was determined based on a previous study of the partner effect^1^. In an experiment with a physically absent partner, one-way ANOVAs on sensitivity (d′) and response time (RT) produced effect sizes (partial *η*^2^) of 0.30 and 0.45, respectively. Taking partial *η*^2^ = 0.3, a power analysis using G*Power 3.1.9.4^12^ yielded a sample size of 18 for an F-test (ANOVA, repeated measures) to achieve a power of 0.90 with an alpha value of 0.05. In reality, to balance the stimulus design, 24 university students in China Medical University, Taiwan, participated in this study (5 males and 19 females). All participants had normal or corrected to normal vision. They provided written informed consent before participating and were debriefed after the session. This study was approved by the Ethics Committee of the China Medical University, and the experiment was carried out in accordance with the Code of Ethics of the World Medical Association (Declaration of Helsinki).

### Stimuli

The task required the participants to associate three geometric shapes-square, circle, and triangle-with three names; the association between shapes and names was counterbalanced across participants. The names were the participant’s best friend’s name, their assigned partner’s name, and a neutral name (a stranger’s). In other words, the task was a within-subject design with 3 identities (friend/partner/stranger) × 2 trials type (match/mismatched).

**Figure 1:**
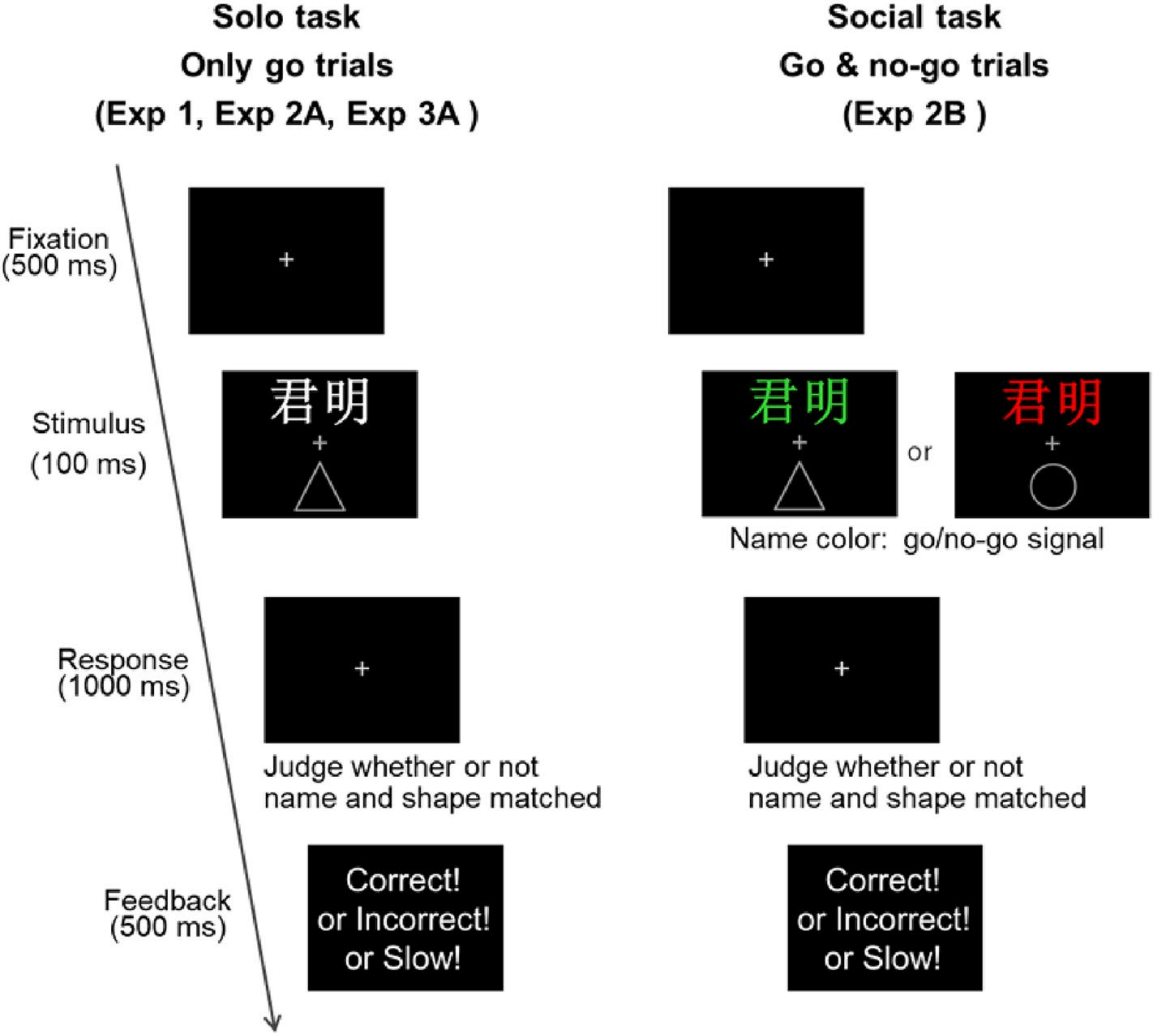
Experimental procedure of solo and social tasks.

To control the length of the names, all consisted of two Chinese characters. If a participant’s name had three characters, the last two characters (i.e., the first or given name) were used. We selected two sets of unisex names for the partner’s/stranger’s names: 佳瑶/君明 (*Jia Yao/Jun Ming*), and 宜詠/逸晴 (*Yi Yong/Yi Qing*). For counterbalancing, half of the participants were given 佳瑶/君明 (*Jia Yao/Jun Ming*) and the other half were given 宜詠/逸晴 (*Yi Yong/Yi Qing*) as the partner’s/stranger’s names. All participants confirmed that before the experiment they did not know anyone with the partner’s/stranger’s names. (If participants had known one of these two names, it would have been replaced with one of the backups: 均銘 (*Jun Ming*), 呈軒 (*Cheng Xuan*), 意晴 (*Yi Qing*), or 逸晨 (*Yi Chen*). The stimuli were shown against a black background. As shown in Fig. 1, one name in white color (visual angle 2.98° × 3.06°) was presented above a central grey fixation cross (visual angle 0.51° × 0.51°), and one grey shape (visual angle 3.82° × 3.82°) was presented below the fixation cross. The distance between the names or shapes and the fixation was a visual angle of 8.89°. The experiment was conducted using E-Prime Professional 2.0 on a 24-inch Dell monitor (E248WFPb, 1440 × 900, 60 Hz).

### Procedure

Upon arrival, the participants were made to believe that they were going to perform a task individually and then together with a partner in a memory experiment on social relationships. They were first asked to provide the name of their best friend and to complete a brief questionnaire about history of their friendship with this selected friend. This procedure was introduced to enrich memories of their best friend^1^. While participants were being given the consent forms to read and sign, the experimenter introduced a confederate who pretended to be the task partner. Participants were not given any opportunity to interact with the partner. Then participants were told that they would perform the individual condition first, and a joint task would follow. In reality, they performed only the individual condition and did not perform the joint task (i.e. the participant performed half of the experiment they were initially told). After the individual condition was completed, we debriefed the participants by revealing the purpose of the research.

Each participant was instructed to remember the matching rules for the three names and three shapes, presented to them on a display monitor. The name-shape association was counter-balanced between participants. Each participant performed 30 practice trials on the name-shape associations before completing three blocks of the solo condition (150 trials per block). The entire experiment lasted about 30 min.

Figure 1 (left panel) summarizes the experimental procedure. All trials began with a fixation cross presented for 500 ms, followed by the name-shape pairing stimulus for 100 ms. Participants responded to all trials (i.e., all were go trials in a solo task). The participants were instructed to indicate as accurately and quickly as possible whether or not the name and the shape matched by using two keys (‘n’ or ‘v’ for one group of participants, ‘1’ or ‘3’ for another) on their own computer keyboard. After the response was received, visual feedback (“Correct” or “Incorrect”) was displayed for 500 ms. If the participant did not respond within 1000 ms, the program displayed feedback consisting of the word “Slow” for 500 ms, and the next trial began. Slow trials were considered inaccurate responses during data analysis. There was no special reward for any specific identity/shape category (friend, partner, stranger), thus ensuring that the participants would not strategically favor any particular identity to maximize the reward.

### Results

The results obtained from all 24 participants were included in the data analysis. Responses faster than 200 ms were excluded, eliminating 5.2% of the trials. Only accurate trials were included in the RT analysis; 11.9% of the trials had erroneous responses and were excluded.

To investigate identity-related advantages, we compared d prime (*d*′) and reaction time (RT) on trials relating to different identities by means of ANOVA. We assess both *d*′ and RT to detect possible speed-accuracy tradeoff (faster response but lower accuracy). After excluding the speed-accuracy tradeoff, we define the partner-advantage if a participant’s RT is significantly lower or the accuracy is significantly higher in the partner-associated trials than that in the stranger-associated trials. We provided RT results from both matched and mismatched trials in our graphs, but we can only perform RT analysis on the matched trials because mismatched trials involve two identities (e.g. friend-related shape with partner’s name) and are hard to be classified.

Accuracy and individual d′ data were also analyzed; these results are provided in the Supplementary Figures for reference. Because *d′* (the difference between z scores of hit and false alarm rates) encompasses both hit rate and false alarm rate, it reflects sensitivity of responses to different identity-shape pairs and was used as an index of performance in previous research on identity referential advantages^6,13^. We considered the presence of an advantage effect to be reflected in either *d*′ or response time.

**Figure 2:**
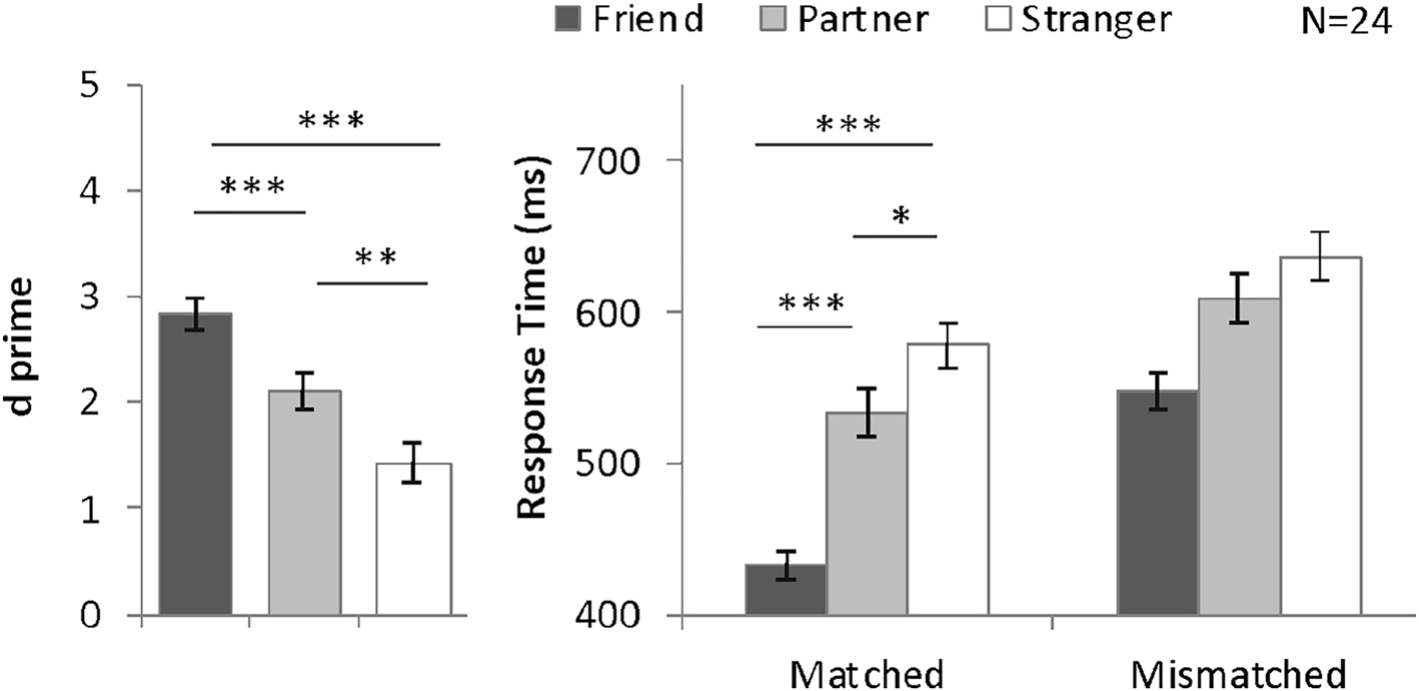
Mean and SE of *d*′ and RT for different shape categories in Experiment 1. *P < 0.05, **P < 0.01, ***P < 0.001.

For the sensitivity analysis, we employed a signal detection approach and calculated *d*′ from both matched and mismatched trials (defined by shape). Extreme hit rates and false alarm rates were adjusted using the log-linear correction method^14^. The *d*′ values for individual participants are shown in Supplementary Fig. 5, and the average results are summarized in Fig. 2. Average accuracy results are included in Supplementary Fig. 1 for reference. A one-way ANOVA on *d*′ with shape category (friend-, partner-, and stranger-associated) as the within-subjects factor revealed a significant main effect, *F*(2, 46) = 29.16, *P* < 0.001, *η*^2^ = 0.56. A planned comparison test with Bonferroni adjustment yielded a greater *d*′ on friend-related trials (*d*′ = 2.83) than on partner-related trials (*d*′ = 2.10, *P* < 0.001) and stranger-related trials (*d*′ = 1.43, *P* < 0.001). Furthermore, participants performed more accurately on partner-related trials than on stranger-related trials (*P* = 0.009). The above results indicated that, when compared with stranger-related trials, both friend-related trials and partner-related trials were processed more accurately under the solo task condition when a joint action was not expected (Fig. 2). We calculate Cohen’s *d*_*rm*_^15^ for planned pairwise comparisons with Bonferroni adjustments in the above repeated measure ANOVA. The effect size results of all experiments are summarized in Table 1. Cohen’s d_rm_ of friend-advantage (1.64) was more than twice as that of Cohen’s drm partner-advantage (0.74).

$$Cohen\text{'}s d_{rm} = \frac{{Mean_{1} - Mean_{2} }}{{\sqrt {SD_{1}^{2} + SD_{2}^{2} - \left( {2 \times r \times SD_{1} \times SD_{2} } \right)} }} \times \sqrt {2\left( {1 - r} \right)} ,$$

where r is the correlation coefficient of the two groups.

**Table 1:** Planned pairwise test results (*p* values and Cohen’s *d*_*rm*_ effect sizes) with Bonferroni adjustments on *d*′ and RT of matched trials for all experiments.

*P* value/	Friend-advantage (Friend vs. stranger)		Partner-advantage (Partner vs. stranger)	
Cohen’s *d*_*rm*_	d′	RT	d′	RT
1	1.64***	2.37***	0.74**	0.68**
2A	1.21***	1.74***	0.50*	0.48
2B	2.05***	1.92***	0.67*	0.76**
3	0.91**	1.34***	0.36	0.12

Friend-advantage refers to friend-related trials versus neutral/stranger trials. Partner-advantage refers to partner-related trials versus neutral/stranger trials. Significant results are shown in asterisks (*): *P < 0.05. **P < 0.01. ***P < 0.001.

Significant values are in [bold].

The results for response time are summarized in Fig. 2. A two-way ANOVA with within-subjects factors of shape category (friend-, partner-, and stranger-associated) and matching judgment (matched and mismatched) revealed significant main effects of shape category, *F*(2, 46) = 72.22, *P* < 0.001, *η*^2^ = 0.76, and matching judgment, *F*(1, 23) = 189.94, *P* < 0.001, *η*^2^ = 0.89. The interaction effect was also significant, *F*(2, 46) = 9.42, *P* = 0.02, *η*^2^ = 0.29. On the matched trials, a significant main effect of shape category was observed, *F*(2, 46) = 60.98, *P* < 0.001, *η*^2^ = 0.73. Planned comparison tests indicated that friend-related trials (433.25 ms) and partner-related trials (533.90 ms) were responded to more quickly than stranger-related trials (578.10 ms; *P* < 0.001 and *P* = 0.02, respectively). Friend-related trials were significantly faster responded than partner-related trials (*P* < 0.001) in matched trials. The effect size of partner-advantage and friend-advantage were calculated and summarized in Table 1. Cohen’s *d*_*rm*_ of friend-advantage (2.37) was more than 3 times larger than that of Cohen’s *d*_*rm*_ partner-advantage (0.68).

The results of Experiment 1 suggest that the task setting of joint action is not necessary to observe the partner effect, i.e. partner-advantage can be observed in a solo-task context also. As long as the identity itself is socially significant (e.g., a partner in a later joint task), the effect is observable in a shape-matching task. This finding is consistent with the observation in Cheng and Tseng’s research in 2019^1^, that is, the physical presence of a partner in a later joint task was critical for this information priority effect. In Experiment 2A, there was no confederate present during the experiment, although a partner’s name was introduced. Again, the participant performed a solo task (i.e., in non-social context).

## Experiment 2A: Solo task with absent social partner

### Purpose

Experiment 2 was designed to investigate whether a partner who did not appear physically and did not speak was sufficient to induce beneficial partner-associated processing in a solo task (2A) and in a social task (2B). The social task (Fig. 1 right panel), adopted from Cheng & Tseng’s research 2019^1^, was a go/no-go task in that the participant was asked to respond to trials with a specific color and was told that the other half trials would be completed by a partner.

### Participants

Another 24 university students participated in this study (9 males and 15 females), all with normal or corrected to normal vision. They provided written informed consent and were debriefed after the session. This study was approved by the Ethics Committee of the China Medical University, and the experiment was carried out in accordance with the Code of Ethics of the World Medical Association (Declaration of Helsinki).

### Stimuli and procedure

All stimuli and procedures were identical to Experiment 1 with the following exceptions: the partner was absent, and only one set of unisex names (君明 and 逸晴) for partner’s and stranger’s names were counterbalanced across participants. The task was a within-subject design with 3 identities (friend/partner/stranger) × 2 trials type (match/mismatched). Participants were told to proceed with the individual condition first, because the partner was in the restroom. In reality, participants performed only the individual condition (solo task) and never met the partner. After the individual condition was completed, participants were informed of the purpose of the research. The participants received instructions and practiced 30 trials before three blocks of the individual condition. The entire experiment lasted about 30 min.

### Results and discussion

**Figure 3:**
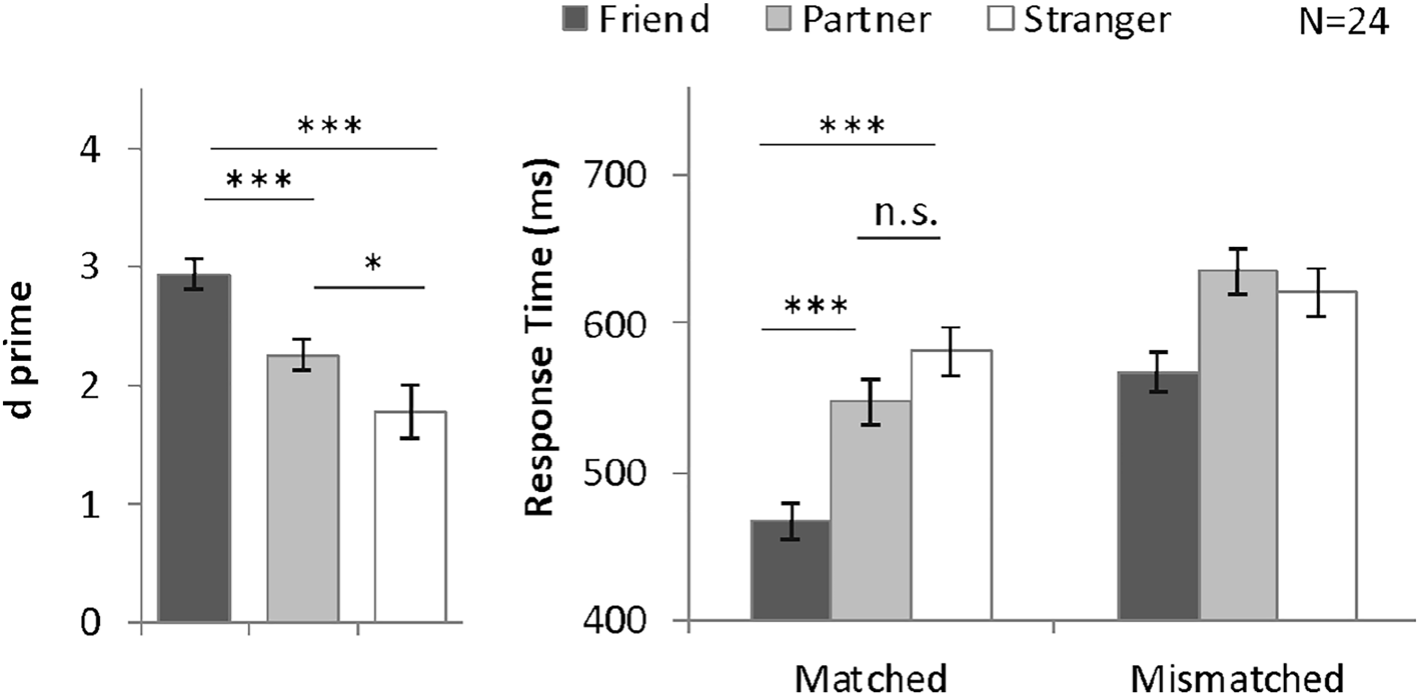
Mean and SE of *d′* and RT for different shape categories in Experiment 2A. *P < 0.05, **P < 0.01, ***P < 0.001.

The results obtained from all 24 participants were included in the data analysis. Responses faster than 200 ms were excluded, thus eliminating 4.9% of the trials. Only accurate trials were included in the RT analysis; 10.4% of the trials had erroneous responses and were excluded. The *d*′ values for individual participants are shown in Supplementary Fig. 6, and the average results are summarized in Fig. 3 and Table 1. Average accuracy results are included in Supplementary Fig. 2 for reference. A one-way ANOVA on *d*′ with the within-subjects factor of shape category (friend-, partner-, and stranger-associated) revealed a significant main effect, *F*(2, 46) = 23.11, *P* < 0.001, *η*^2^ = 0.50. Planned comparisons with Bonferroni adjustments indicated greater *d*′ on friend-associated trials (*d*′ = 2.94) than on partner-associated (*d*′ = 2.26, *P* < 0.001) as well as stranger-associated trials (*d*′ = 1.78, *P* < 0.001). Further, participants performed more accurately on partner-associated trials than on stranger-associated trials (*P* = 0.04). These results indicate that stimuli associated with friends and partners were processed more accurately than with strangers under the solo task condition when a joint action was expected later (Fig. 3). The Cohen’s *d*_*rm*_ of friend-advantage (1.21) was more than twice as much as partner-advantage (0.50).

The response time results are summarized in Fig. 3. A two-way ANOVA with within-subjects factors of shape category (friend-, partner-, and stranger-associated) and matching judgment (matched and mismatched) revealed significant main effects of shape category, *F*(2, 46) = 42.96, *P* < 0.001, *η*^2^ = 0.0.65), and matching judgment, *F*(1, 23) = 132.28, *P* < 0.001, *η*^2^ = 0.85. The interaction effect was also significant, *F*(2, 46) = 7.93, *P* = 0.001, *η*^2^ = 0.26. On the matched trials, a significant main effect of shape category was observed, *F*(2, 46) = 35.74, *P* < 0.001, *η*^2^ = 0.61. Planned comparisons indicated that friend-associated trials (466.98 ms) elicited significantly quicker responses than partner-associated (547.44 ms, *P* < 0.001) and stranger-associated trials (581.47 ms, *P* < 0.001). There was no significant difference between partner-associated trials and stranger- associated trials (*P* = 0.08).

The results of Experiment 2A suggest that a partner, even one who does not appear physically, still generates priority processing. This is surprising, because Cheng and Tseng^1^ found that a partner who did not appear failed to provide this endorsement. There are two major differences between the present experiment and Experiment 3 of Cheng and Tseng’s 2019 study^1^: the present experiment has only go trials (instead of both go and no-go trials), and use friend as the reference identity (instead of self). To identify which factor is the more likely reason for this advantage, we repeated Experiment 2A with both go- and no-go trials, as in Experiment 3 of Cheng and Tseng’s 2019 study^1^, while keeping friend as the reference identity.

## Experiment 2B: Social task with absent social partner

### Purpose

This experiment was designed to investigate whether a partner who did not appear physically and did not speak was sufficient to induce beneficial partner-associated processing in a social task.

### Participants

Another 24 university students participated in this study (12 males and 12 females), all with normal or corrected to normal vision. They provided written informed consent and were debriefed after the session. This study was approved by the Ethics Committee of the China Medical University, and the experiment was carried out in accordance with the Code of Ethics of the World Medical Association (Declaration of Helsinki).

### Stimuli and procedure

The procedures and the stimuli were identical to those used in Experiment 2A with the following exceptions: First, the number of trials was doubled, with half of the trials appearing in red on the display and the other half in green (see Fig. 1, social task). Each participant was instructed to respond to green trials only (go trials) but not the red trials (no-go trials) because their partner, waiting in another room, would respond to the red trials afterwards. In reality, each participant performed 30 practice trials, followed by three blocks alone (individual condition) and a debriefing session. The entire experiment lasted about 45 min.

### Results and discussion

The results obtained from all 24 participants were included in the data analysis. Responses faster than 200 ms were excluded, thus eliminating 3.9% of the trials. Only trials with correct responses were included in RT analyses; 8.8% of the trials had incorrect responses and were excluded.

**Figure 4:**
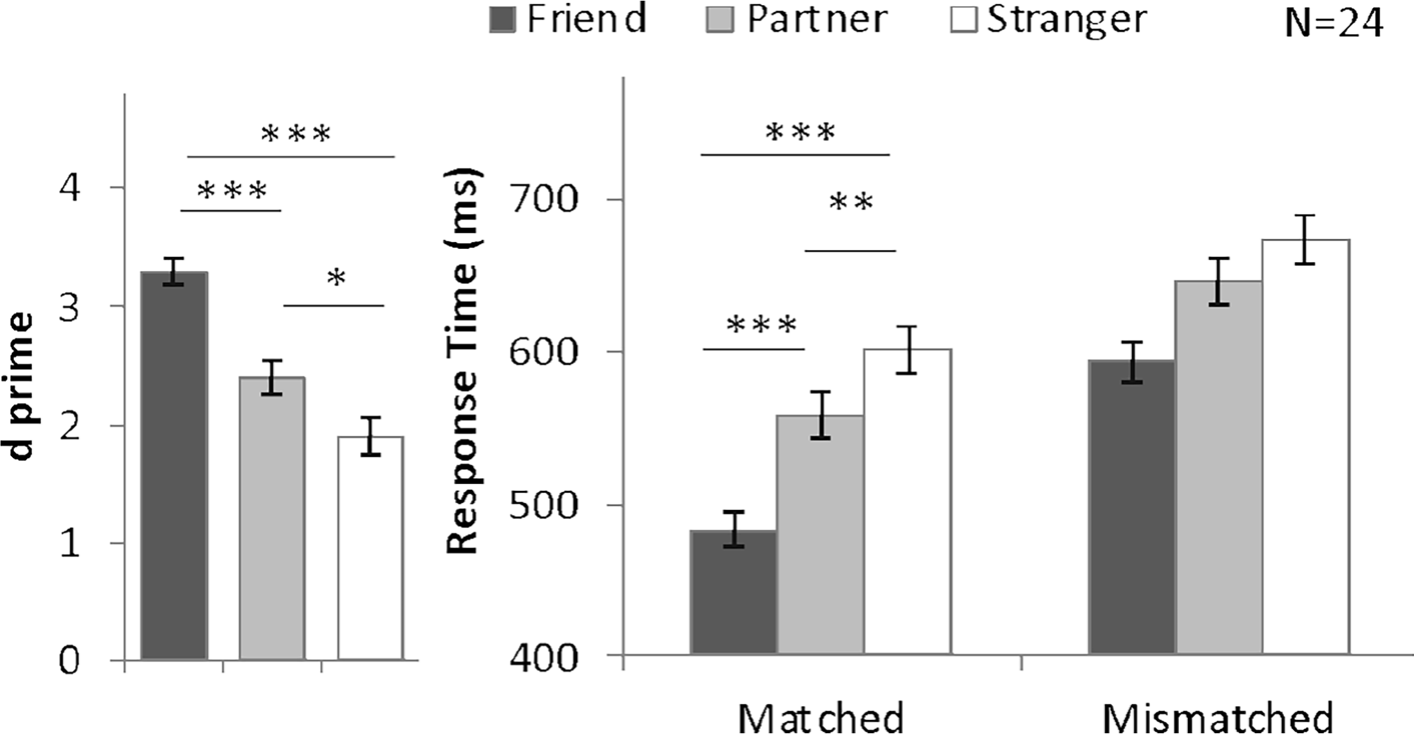
Mean and SE of *d′* and RT for different shape categories in Experiment 2B. *P < 0.05, **P < 0.01, ***P < 0.001.

As in Experiment 1, ANOVAs were conducted on *d′* (Fig. 4) and RT (Fig. 4) to investigate identity-associated advantages, and Cohen’s *d*_*rm*_ in Table 1. The *d′* values of individual participants are shown in Supplementary Fig. 7. A one-way ANOVA on *d′* indicated a significant main effect of shape category, *F* (2, 46) = 36.04, *P* < 0.001, *η*^2^ = 61. Planned comparisons revealed greater *d*′ for friend- (3.30, *P* < 0.001) and partner-associated stimuli (2.39, *P* = 0.03) than for stranger-associated stimuli (1.89). In addition, friend-associated shape showed higher *d*′ than partner-associated shape (*P* < 0.001). The Cohen’s *d*_*rm*_ of friend-advantage (2.05) was more than twice larger than partner-advantage (0.67) (Table 1). Average accuracy results are included in Supplementary Fig. 3 for reference.

A two-way repeated-measures ANOVA on response times with the factors of shape category and matching judgments revealed a significant interaction effect, *F*(2, 46) = 4.32, *P* = 0.02, *η *^*2*^ = 0.16. Planned one-way ANOVAs applied to matched trials separately revealed a significant main effect of shape category on matched trials, *F* (2, 46) = 53.91, *P* < 0.001, *η*^2^ = 0.70, such that friend-associated trials (483.05 ms) and partner-associated trials (558.26 ms) were responded to significantly more quickly than stranger-associated trials (600.82 ms; *P* < 0.001, Cohen’s *d*_*rm*_ = 1.92 and *P* = 0.006, Cohen’s *d*_*rm*_ = 0.76 respectively).

Our finding suggests that the partner-advantage is susceptible to stimulus design and more visible when the task does not involve self-identity. One noteworthy observation is that the advantage effect occurred in the absent partner condition of Experiment 2B, but did not appear in our previous study (Experiment 3, Cheng & Tseng, 2019^1^). The major difference in the experiment design between the two experiments was that Cheng and Tseng^1^ used self as a reference identity (the identities were self-partner-stranger) and the present study replaced self with the best friend. As the ceiling effect is commonly observed in the self-related trials, it is possible that partner-advantage is easier to be observed when a less dominant identity is involved (e.g. replace self with friend). One possibility is that the self is a dominant identity that consumes considerable cognitive resources, there may be little left for other competing identities (i.e., the partner) in these tasks. Similarly, other studies have reported that friend bias is smaller than self-bias and sometimes disappears^6,9,16,17^. How reference identities anchor the occurrence and size of the partner-effect is worth further investigation.

The results of Experiment 2A (solo task) and 2B (joint task) show a consistent processing advantage for an absent partner, suggesting that physical presence is not a necessary condition for partner prioritization. Other factors, such as reference identities (e.g., self, best friend), may play a bigger role. Together, Experiments 1 and 2 suggest that visual presence is not a must: A partner in a joint task benefited from a social advantage that did not accrue to the neutral stranger’s name. The cause of this facilitation remains unclear: Is a co-working relationship required, or is this a general effect of affiliation after initial social contact? In Experiment 3, we tested if the partner label is a sufficient condition for the advantage effect by placing a co-working partner label on a non-partner person.

## Experiment 3: Solo task with absent non-partner (another participant)

### Purpose

This experiment investigates whether an identity advantage toward a non-partner (another participant) is present with an initial affiliation that does not lead to a future co-working relationship.

### Participants

Twenty-four students who did not participate in the previous experiments took part in this study (9 males and 15 females). All had normal or corrected to normal vision, provided written informed consent, and were debriefed at the conclusion of the study. This study was approved by the Ethics Committee of the China Medical University, and the experiment was carried out in accordance with the Code of Ethics of the World Medical Association (Declaration of Helsinki).

### Stimuli and procedures

All stimuli and procedures were identical to Experiment 1 except that participant were told to associate three shapes with their friend’s name, another participant’s name, and a stranger’s name, respectively. The task was a within-subject design with 3 identities (friend/another participant/stranger) × 2 trials type (match/mismatched). The partner names used in Experiment 1 were now being instructed as another participant in the current experiment. There was no joint task or partner expected in this experiment. The participants completed 30 practice trials before three experimental blocks. Eight participants practiced twice because of difficulty understanding the instructions or poor performance on the practice trials (over 30% in error rate). The entire experiment lasted about 30 min.

### Results

The results obtained from all 24 participants were included in the data analysis. Responses faster than 200 ms were excluded, thus eliminating 9.9% of the trials. Only accurate trials were included in the RT analysis; 8.5% of trials were thus excluded.

**Figure 5:**
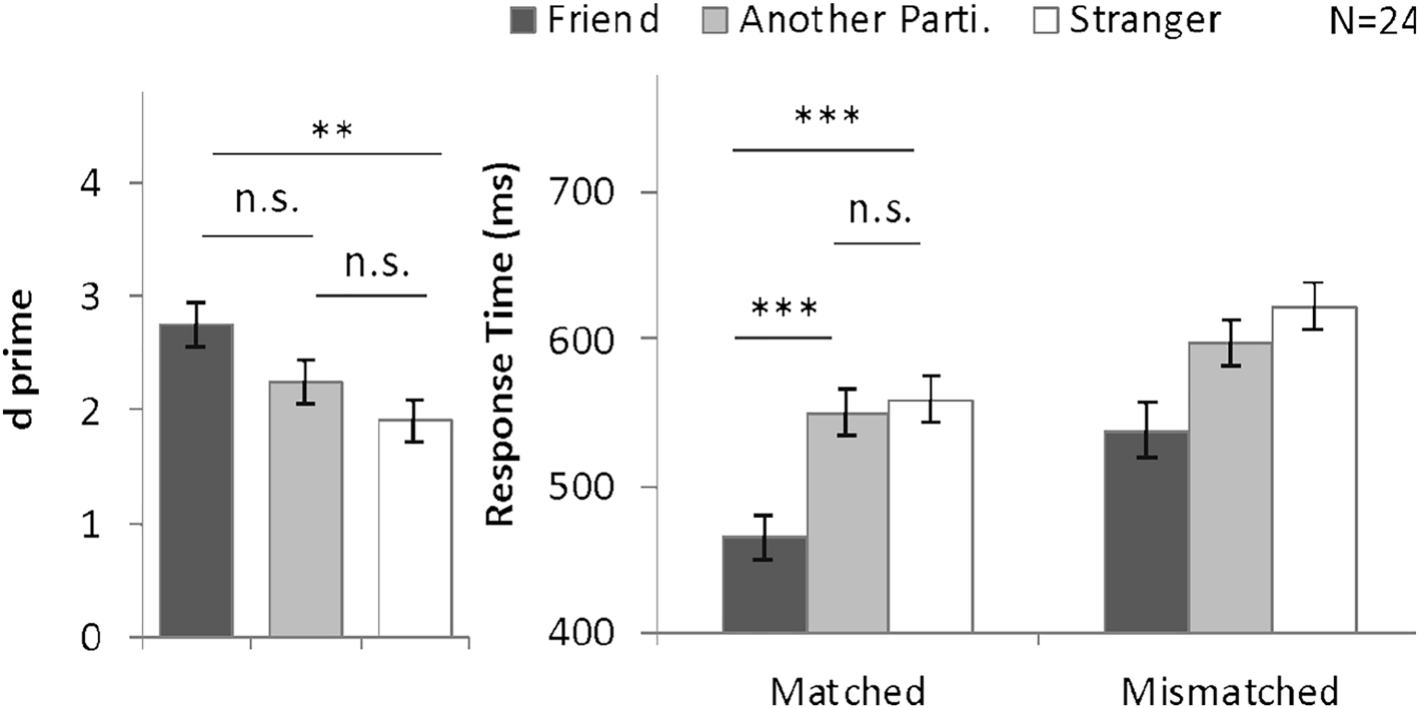
Mean and SE of *d′* and RT for different shape categories in Experiment 3. *P < 0.05, **P < 0.01, ***P < 0.001.

To investigate identity-related advantages, we compared *d*′ and RT on trials relating to different identities with ANOVAs (Fig. 5), and Cohen’s *d*_*rm*_ in Table 1. The *d*′ values for individual participants are shown in Supplementary Fig. 8. For reference, Supplementary Fig. 4 displays average accuracy by blocks. A one-way ANOVA on *d*′ with the within-subjects factor of shape category (friend-, partner-, and stranger-associated) revealed a significant main effect, *F*(2, 46) = 9.76, *P* < 0.001, *η*^2^ = 0.30. Planned comparisons with Bonferroni adjustments indicated greater *d′* on friend-associated trials (*d*′ = 2.75) than on stranger-associated trials (*d′* = 1.91; *P* = 0.002; Cohen’s *d*_*rm*_ = 0.91), while *d′* was similar for trials associated with another participant (*d*′ = 2.24) and for those associated with strangers (*P* = 0.12; Cohen’s *d*_*rm*_ = 0.36).

The response time results are summarized in Fig. 5 (right panel). A two-way ANOVA with within-subjects factors of shape category and matching judgment (matched and mismatched) revealed significant main effects of shape category, *F*(2, 46) = 49.20, *P* < 0.001, *η*^2^ = 0.68, and matching judgment, *F*(2, 46) = 48.00, *P* < 0.001, *η*^2^ = 0.68). The interaction effect was not significant (*P* = 0.25). Although the interaction was not significant, we still conducted planned follow-up ANOVA on the matched trials. Results showed a significant main effect of shape category, *F*(2, 46) = 31.27, *P* < 0.001, *η*^2^ = 0.58. Planned comparisons indicated an advantage for the friend over strangers (464.44 ms vs. 558.96; *P* < 0.001; Cohen’s *d*_*rm*_ = 1.34) but not for the other participant over strangers (550.03 ms vs, 558.96 ms; *P* > 0.99; Cohen’s *d*_*rm*_ = 0.12). And the response time for friend-related trials was shorter than partner-related trials (*P* < 0.001). The above results indicate that when compared with strangers, the other participant did not enjoy a processing benefit under the solo task setting (Fig. 5).

The results of Experiment 3 are important in several ways. First, when names were introduced as participants in the same experiment, they did not automatically benefit from a processing advantage. Thus the partner bias observed in cooperative relationships^1^ may not be a general effect that is applicable to other social categories (e.g., in-group membership). Therefore, our results identified one of the boundary conditions of the partner bias. Second, when considered together with the results of Experiment 2, our results demonstrate that physical presence is not a prerequisite to formation of a perceptual priority, even for a new acquaintance. Factors such as social affiliation, social significance, or social context may be weighted more in forming a perceptual priority.

## General discussion

We investigated the sufficient conditions of the partnership advantage in four experiments. We manipulated the social nature of the task by including a partner (social affiliation) or by applying a joint go/no-go task (social context). We observed priority processing toward an arbitrary shape associated with the partner identity in all conditions (Experiments 1 and 2), except when the both partner label and social context were removed (Experiment 3). Our results suggest that the social affiliation generated by a co-participant is necessary and sufficient to establish a partner-advantage, even in a solo-task context. On the other hand, a social joint task is not a pre-requisite to observe the partner-advantage.

Although partner bias has been reliably demonstrated with both human partners^1^ and robot partners^18^, the necessary and sufficient conditions to generate and maintain this effect have not been fully understood. Constable and colleagues^8^ tested the partner-advantage with a similar go/no-go identity-shape matching task. Participants were asked to associate five shapes to self, partner (co-actor), stranger, we-team, and stranger-team. They were tested in pairs, and within a pair, one participant was responsible for responding to matched trials (the identity matched the shape) while the other was responsible for responding to mismatched trials (the identity did not match the shape). While self-related trials enjoyed a reliable self-bias, partner-related trials did not incur processing advantages over the stranger-related trials. There are several possible factors that precluded partner bias in their study. First, the cognitive loads required to memorize the 5-pair matching rules in Constable and colleagues’ study^8^ were greater than those needed for the 3-pair matching rules in our studies. When multiple identities compete for limited cognitive resources, the partner-advantage may become unstable and less readily visible when it confronts other more robust identities, such as self. Second, how the joint task is devised may also modulate the partner effect. In our joint task, participants divided the trials by color (red or green), and each participant responded to an equal number of trials. Their task was to judge whether trials for which they were responsible contained matched or mismatched shape identities. In Constable and colleagues’ study^8^, the participants in a pair were instructed to respond to either the matched or mismatched trials. This task design had two immediate consequences: (1) the effort required to perform the shared task was unequal, as the mismatched trials always required longer reaction times; (2) the identity biases (e.g., self-bias, we-bias) appeared only on the matched trials, which were responded to by one participant. The other participant, who was responsible for the mismatched trials, failed to show even self-bias. This suggests that the nature of the divided tasks differs greatly. Further, it is unclear whether matched trials and mismatched trials generated a similar co-representation beneficial to the social valence for partners. To sum up, the factors that modulate the short-lived partner bias remain unclear, and a systematic and parametric future study is warranted.

What are the underlying mechanisms that serve the partner-advantage? Is it produced on the same pathway as other identity-related biases, such as the self-advantage and the friend-advantage? Or is it served by a distinct pathway, one that is related or unrelated to the source of identity-related biases? Available evidence suggests that there are a few distinct differences between partner-advantage and self-advantage/friend-advantage. First, previous research implies that long-term memory enhancement may play a different role to exert the partner-advantage, compared to self-advantage. Studies of self-referential processing have suggested that self-reference facilitates memory for neutral stimuli^19–21^, and the memory enhancement is more robust with self-reference than with other identities. Wang et al.^16^ reported a switch cost for self-related shapes. When self-related shapes were reassigned to new identity labels, participants made more errors and slower responses on trials with shapes previously associated with self, demonstrating the difficulty of unbinding self-related associations in memory. However, this may not be the primary account of the partner-relevant advantages. A similar switch cost was not observed for partner-related shapes. Constable and Knoblich^22^ incorporated a joint task and a new “partner” into their experimental design to investigate the memory hypothesis of the identity-prioritization effect. When the identity-shape pair was switched after the participants were familiarized with the pairing, a switch cost to self-related trials was incurred; that is, the response times to the new shape associated with self became significantly longer than response times to the old shape. Interestingly, the rematched partner shape did not have switch cost, it even generated the opposite effect (i.e., a learning effect). This suggests that the referential association of partner is easier to be detached and reassigned to another identity, possibly not originating primarily from the memory enhancement. However, it is important to note that the study design used by Constable and Knoblich^22^ differed from our designs in many ways, as described above, and has not always been successful in replicating the partner-advantage (e.g., Constable and colleagues^8^). Thus it may be inappropriate to directly generalize their findings to the present study.

Further, the occurrence of partner-advantage is vulnerable to paradigm design, while self-advantage remains dominant across different task settings and stimulus designs. As discussed above, partner-advantage is absent if a joint task includes no-go trials and self is included as a reference stimulus (Experiment 3, Cheng & Tseng, 2019^1^), or if the shape-matching task contains more identities (five identities in Constable & Knoblich, 2020^22^). An extensive literature has revealed that the identity processes related to significant others are more malleable than self-advantage. For example, the friend-advantage disappeared when the friend was associated with low reward and the stranger was associated with high reward^9^, and the mother advantage disappeared when the probability of occurrence of mother-related stimuli was lowered^10^. On the other hand, self-advantage persisted with these manipulations of Sui and colleagues. The potency of self-advantage is an affirmative support of the binary view of identity-related processing: self is different from others. Our study opens a new question: is all other-related processing the same? Combining our findings and other research, partner-advantage is more vulnerable than significant other advantage, in terms of smaller effect size (see Cohen’s *d*_*rm*_ results in Table 1 in current study; and Table 2 in Cheng & Tseng, 2019), and shorter lifetime (partner advantage could disappear after 300 trials in Cheng & Tseng, 2019). This poses a theoretical challenge to the dichotomous framework of identity referential processing. That is, the binary view of self versus non-self may need to be re-evaluated, at least for the processing related to others. An analogous perspective that includes other governing factors (signification, memory, etc.) may better serve as a more comprehensive model that accounts for both significant others and a newly met partner.

It is important to note the recent resurgence of the view of self-bias effects as distinct processes across cognitive domains (e.g., memory, attention, perception) rather than a common phenomenon^23^. The low-level, automatic responses to self-relevant cues usually result in a positive endowment, which may carry an evolutionary benefit in maintaining self-esteem^24^. Self-related stimuli also alter our attentional selection processes, such as the recovery window needed in a rapid serial visual presentation task (i.e., the attentional blink). When participants hear their own name, it is less susceptible to interference from other items because of its high social salience^3^. Although these observations all signify the importance of self-construal, the empirical data show little correlation between the effect sizes across tasks that tap different cognitive domains. This leads to a call to evaluate self-processing with a diverse rather than a unified network. Little is known about whether other-related processing can also be viewed from a similar, distributed perspective. Even the most robust identity-referential advantage, the self-advantage, shows a gradient in intensity that can be adjusted according to temporal and identity relevance to the current self. For example, self-advantage measured in a blocked design (one shape and all three identities presented) is lower than self-advantage measured in an intermixed design (all three shapes and all three identities presented^17^). It has been speculated that self-advantage weakens because participants habituate to the self-shape presented on every trial in a block design, resulting in reduced saliency and impaired working memory for the shape-label association^25–28^. As the self is an identity we carry since birth, Golubickis et al.^29^ tested shape-identity matching performance for current self, future self, past self, and stranger. Only the current self was advantageous over the stranger. This signifies the multifaceted dimension of the self.

The extensive interplay between self and others in shape-matching tasks suggests that the mechanisms underlying self-advantage, friend-advantage, and partner-advantage are probably interweaved and modulated by relevance to self. In Golubickis and colleagues’ study in 2020^30^, participants rated membership categories (i.e., vegetarian, athlete, musician) and personality traits (i.e., Big Five) as high, medium, or low in relevance to themselves. In subsequent shape-information matching tasks, shapes associated with low self-relevant information showed less prioritized processing than high self-relevant information. This suggests that we automatically enter a judgment of proximity to ourselves even when it is not task-related. Moreover, self-prioritization could be generalized to the group “we”. Constable and colleagues’ study in 2019^8^, had participants remember association between four identities (self, we, s/he, and they) and four geometric shapes. Participants performed better on we-relevant stimuli than on they-relevant ones (Experiments 1, 2, 3). To summarize, even for other-category identities, the strength of embedded self-relevance regulates their advantage.

The partner-advantage has an implication in real world communication. We are now more frequently working with people with minimum exposure and prior contact, and the team-formation in the borderless cyber world is quick to build, re-structure, and dissolve. In our experiments, two people assigned to the same organization but did not share works do not enjoy partner-advantage automatically. This signifies that “in-group” and “out-group” social labels^31^ are not the basis of this novel identity preferential processing. The partner-advantage can be observed instantaneously with minimum information about the partner, making it very relevant to the new co-working styles with partners remotely or in the cloud. However, the malleability of this advantage applies well in the modern world where we need to quickly form a team and re-organize our team members for different task demands. These speculations can be tested in future studies. For example, Wang et al.^16^ used the switch-cost on self-related trials to probe the memory enhancement in self-related processing. We hypothesize that the partner-associated trials would produce no switch cost because of its fundamental difference from self-advantage. The definition of “partner” also needs elaboration. We hypothesize that only co-workers who share joint work are endowed with this processing advantage, but not the same-group members who do not share work or have independent work goals. It is also intriguing to further investigate whether a “cooperative partner” and a “competitive partner” exerts the same priority effect in the identity-shape association task. While “partners'' can be understood as the extension of self concept^32,33^ to shares the self-related neural processing in a rapid and flexible manner^34^, it is interesting to ask whether self-relevant identities (both “cooperative” and “competitive” partners are self-relevant) can be further separated by their self-other overlap (“cooperative” partners overlap with the self, but the “competitive” partners do not). Finally, individual differences (e.g. altruists v.s. non-altruistists in Brethel-Haurwitz et al., 2018^35^) were reported to associate with the brain activation related to self and others. Along this dimension, a person’s social tendency (e.g. autistic traits), trustiness, altruistness, or personal responsibility may also contribute to the formation of the partner-advantage.

In summary, our study offers new insights on identity-referential processing. Together with other literature, these results suggest that it is necessary to reconsider the identity-referential advantage along a spectrum from self and significant others to acquaintances and newly met partners.

## Supplementary Information

Supplementary Information.

## Data Availability

The datasets used and/or analysed during the current study available from the corresponding author on reasonable request.
