# Supplementary material for: Social affiliation is sufficient to provoke the partner-advantage
Source: Sci Rep. 2022 Dec 9;12:21293. doi: 10.1038/s41598-022-25052-1 (PMC9734190; doi:10.1038/s41598-022-25052-1)
Supplement: Supplementary file 1 — Supplementary Information. [file 41598_2022_25052_MOESM1_ESM.docx]

**Supplementary materials**

Supplementary Figure 1. Accuracy results in Experiment 1. Mean and SE of accuracy for different shape categories in matched and mismatched trials. **p* < .05. ***p* < .01. ****p* < .001

Supplementary Figure 2. Accuracy results in Experiment 2A. Mean and SE of accuracy for different shape categories. **p* < .05. ***p* < .01. ****p* < .001.

Supplementary Figure 3. Accuracy results in Experiment 2B. Mean and SE of accuracy for different shape categories. **p* < .05. ***p* < .01. ****p* < .001.

Supplementary Figure 4. Accuracy results in Experiment 3. Mean and SE of accuracy for different shape categories. **p* < .05. ***p* < .01.

Supplementary Figure 5.


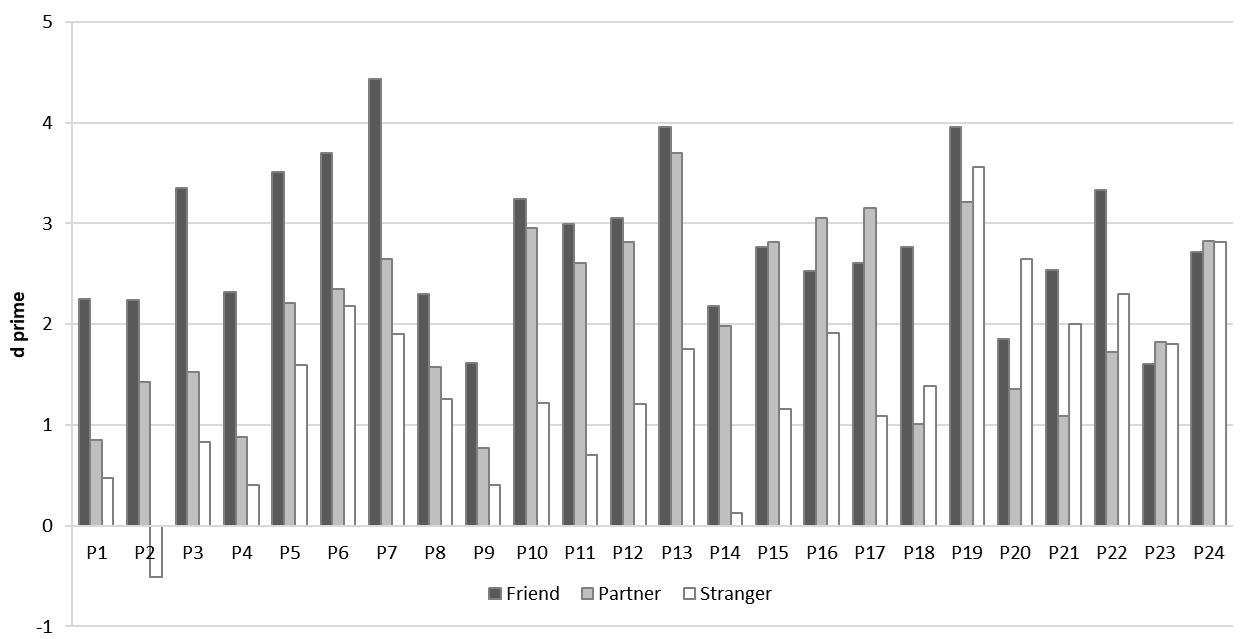


Supplementary Figure 5. *d'* of different shape categories for each participant in Experiment 1. Participants who showed stronger friend- and partner-advantages are on the left and those who showed weaker effects are on the right.

Supplementary Figure 6.


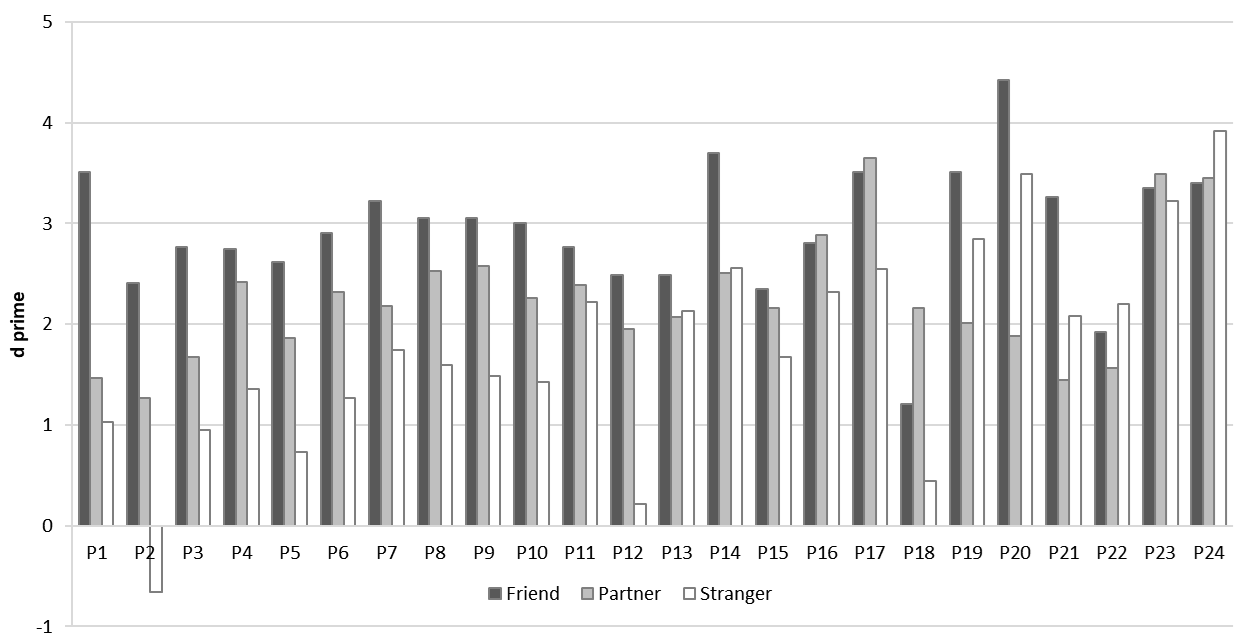


Supplementary Figure 6. *d'* of different shape categories for each participant in Experiment 2A. Participants who showed stronger friend- and partner-advantages are on the left and those who showed weaker effects are on the right.

Supplementary Figure 7.


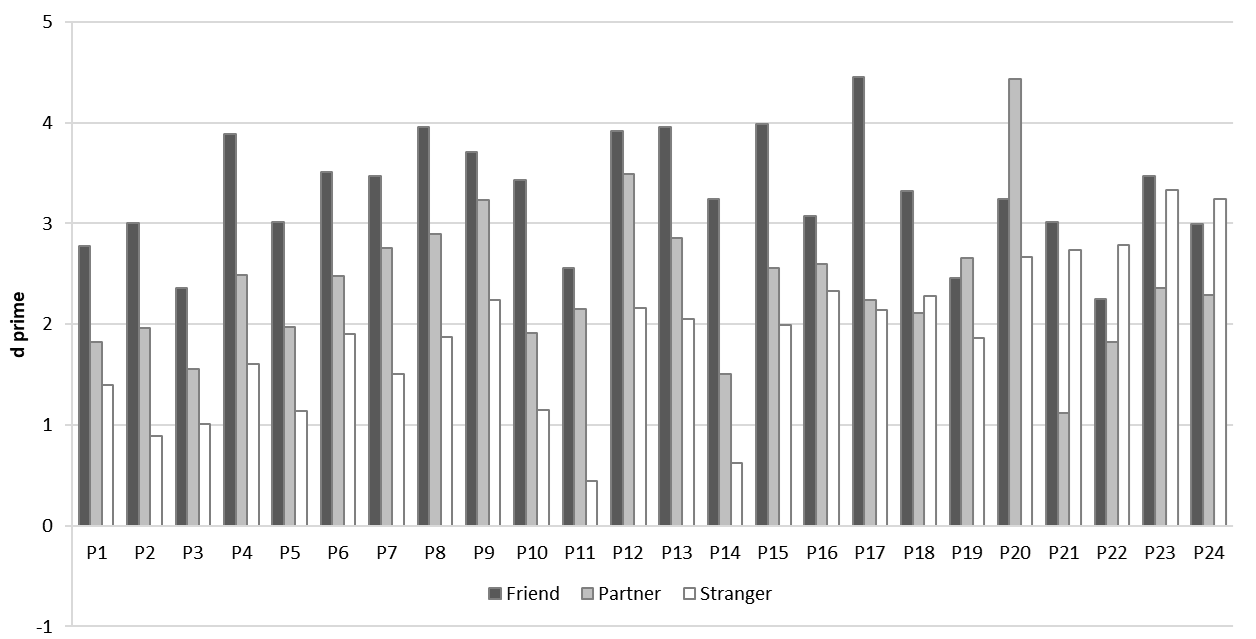


Supplementary Figure 7. *d'* of different shape categories for each participant in Experiment 2B. Participants who showed stronger friend- and partner-advantages are on the left and those who showed weaker effects are on the right.

Supplementary Figure 8.


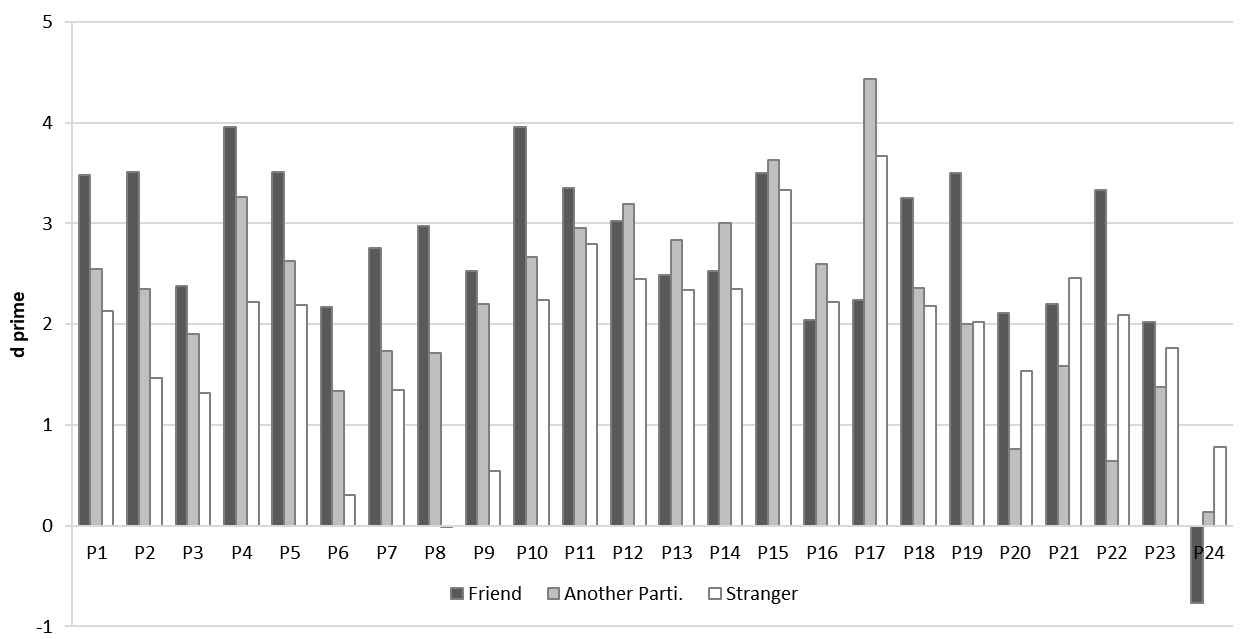


Supplementary Figure 8. *d'* of different shape categories for each participant in Experiment 3. Participants who showed stronger friend- and partner-advantages are on the left and those who showed weaker effects are on the right.
